# Changes in cancer incidence and stage during the COVID-19 pandemic in 2020–2021 in the Nordic countries

**DOI:** 10.2340/1651-226X.2025.42079

**Published:** 2025-02-12

**Authors:** Anna L.V. Johansson, Anna Skog, Tom Børge Johannesen, Tor Åge Myklebust, Simon M. Kønig, Charlotte Wessel Skovlund, Lina Steinrud Mørch, Søren Friis, Marnar Fríðheim Kristiansen, David Pettersson, Eva María Gudmundsdóttir, Nanna Margrét Kristinsdóttir, Helgi Birgisson, Sandra Irenaeus, Johan Ahlgren, Mats Lambe, Elli Hirvonen, Janne Pitkäniemi, Giske Ursin

**Affiliations:** a Cancer Registry of Norway, Norwegian Institute of Public Health, Oslo, Norway; b Department of Medical Epidemiology and Biostatistics, Karolinska Institutet, Stockholm, Sweden; c Department of Research and Innovation, Møre and Romsdal Hospital Trust, Ålesund, Norway; d Danish Cancer Institute, Danish Cancer Society, Copenhagen, Denmark; e Center of Health Science, Faculty of Health Sciences, Tórshavn, Faroe Islands; f National Hospital of the Faroe Islands, Tórshavn, Faroe Islands; g National Board of Health and Welfare, Stockholm, Sweden; h ICS Research and Registration Center, Icelandic Cancer Society, Reykjavík, Iceland; i Regional Cancer Center Central Sweden, Akademiska sjukhuset, Uppsala, Sweden; j Department of Immunology, Genetics and Pathology, Uppsala University, Uppsala, Sweden; k Finnish Cancer Registry, Helsinki, Finland; l Institute of Basic Medical Sciences, University of Oslo, Oslo, Norway; m Department of Preventive Medicine, University of Southern California, Los Angeles, CA, USA

**Keywords:** COVID-19, cancer, incidence, cancer staging, Nordic

## Abstract

**Background and purpose:**

The COVID-19 pandemic impacted substantially on cancer healthcare, including the temporary suspension of screening activities. We compared cancer incidence rates and stage during 2020–2021 to pre-pandemic rates in the Nordic countries.

**Material and methods:**

Using data from the national cancer registries in Denmark, Finland, Iceland, Norway, and Sweden, we estimated age-, sex-, and period-adjusted incidence rate ratios, expressed as relative percentage change (%) with 95% confidence intervals (CIs), comparing rates in 2020–2021 to those in 2017–2019 (pre-pandemic).

**Results:**

In 2020–2021, 340,675 cancer cases were diagnosed. The incidence rates declined during the first pandemic wave (Q2 2020), ranging from –21.7% [95% CI: –23.3%; –20.2%] (Sweden) to –7.9% [–17.7%; 3.0%] (Iceland). Incidence rates also declined in the second pandemic wave (Q1 2021), ranging from –8.6% [–10.2%; –6.9%] (Sweden) to –2.3% [–4.6%; 0.1%] (Norway), and in Sweden also by –3.1% [–4.8%; –1.3%] in the third pandemic wave (Q4 2021). Stage I breast cancer incidence declined during 2020 in Denmark/Norway/Sweden, with some catch-up in stage II incidence in 2021. Prostate cancer rates declined in Denmark/Finland/Norway/Sweden during 2020–2021, while melanoma rates declined in Finland in 2020. During 2020, colon cancer rates declined in Denmark and Iceland, while rectal cancer rates declined in Denmark, and lung and kidney cancer rates declined in Norway.

**Interpretation:**

During 2020–2021, cancer incidence rates declined across the Nordic countries with the largest declines in Sweden. During the third pandemic wave, the incidence rates were mostly similar to pre-pandemic rates. Changes in cancer stage may reflect reduced screening activities.

## Introduction

The COVID-19 pandemic has had a global impact on healthcare with subsequent effects also on non-COVID-19 conditions, such as cancer [1]. Early reports indicated substantial declines in the number of newly diagnosed cancer cases during the first pandemic wave in Spring 2020 [2–4]. These early declines in cancer incidence have been attributed to halted or reduced screening activities, lower screening attendance, and changes in healthcare seeking behavior during lockdowns. In addition, health care was under pressure, with strained resources for diagnostic workup of cancer during pandemic waves [1]. In December 2020, the first severe acute respiratory syndrome coronavirus 2 (SARS-CoV-2) vaccines were introduced to the general public. Since the vaccines mainly targeted severe COVID-19 disease, pandemic mitigation efforts remained in place during parts of 2021, including travel restrictions, bans on large gatherings, and lockdowns.

In the Nordic region, the incidence and mortality rates of COVID-19 differed substantially between countries during the first 2 years of the pandemic. The first wave occurred from March to June 2020, the second from November 2020 to June 2021, and the third from August to December 2021 (Supplemental Figure S1). Sweden had the highest COVID-19 rates during 2020, and by the end of 2020, rates also increased in Denmark. Throughout 2020 and 2021, rates remained substantially lower in Norway, Finland, and Iceland. Testing for SARS-CoV-2 became widely available from July 2020; hence, the numbers of undetected cases were likely highest during the first pandemic wave. Pandemic mitigation efforts were in place in all Nordic countries, including periods of strict lockdown, except in Sweden, where restrictions were primarily implemented in elderly care homes, whereas primary schools and workplaces remained open [5].

During 2020, mammographic screening activities differed between the Nordic countries, from being halted (fully or partially) 3 months or more in Norway, Sweden, and Iceland, to remaining fully operational in Denmark and Finland [6]. However, both Denmark and Finland reported lower attendance rates during 2020. By August 2020, screening was operational in Norway, albeit with a somewhat lower activity than normal, while remaining reduced in Sweden until the end of 2020. Vaccination for SARS-CoV-2 started in December 2020 in the Nordic countries with a very high uptake from the start [7]. By the end of September 2021, 91.2% of the population in Iceland had received at least one dose (71.5% were fully vaccinated), while the corresponding proportions in Denmark were 88.6% (71.5%), Norway 87% (56.6%), Finland 84.2% (50.3%), and Sweden 81.7% (55.6%) [7].

We have previously shown, based on pathology reports only, that the annual number of malignant tumors in the Nordic countries declined substantially in Sweden (–6.2%) and Finland (–3.6%) during the first year of the pandemic 2020 in comparison to 2019 [6]. However, this study did not assess effects across different cancer sites or by stage. In this updated analysis, we used quality-assured data based on all cancer notifications to each Nordic cancer registry to assess whether the cancer incidence rates differed across the Nordic countries during the first, second, and third pandemic waves in 2020 and 2021 compared to pre-pandemic years (2017–2019). We examined all cancers combined, as well as site-specific differences across seven major cancer sites (breast, prostate, melanoma, colon, rectal, lung, and kidney). In addition, we investigated whether stage-specific cancer incidence changed during 2020 and 2021 across the major cancer sites.

## Methods

In this population-based study, we included data of incident cancer cases from the national cancer registries in Denmark, Finland, Iceland, Norway, and Sweden. The Nordic cancer registries obtain cancer notifications from several sources, including clinical and pathological reports, inpatient registries, and death certificates. The different data sources are consolidated at each cancer registry, which help quality-assure the data and ensure timeliness [8]. The national cancer registries have high quality and completeness, with mandatory reporting and 92–97% morphologically verified diagnoses [8, 9]. From each cancer registry, we included all diagnoses of primary cancer from January 2017 to December 2021, without restriction on previous cancer history prior to 2017. To obtain the complete picture of the clinical burden of new cancer cases diagnosed during the pandemic, we did not apply the International Agency for Research on Cancer multiple primary rules to the data. This means that we counted all new cancer cases, even if the patient had previously been diagnosed with a tumor of the same histological group with the same two-digit topography code. Hence, the numbers are slightly higher than those reported in official summary statistics [10]. The cancer registries classify cancer diagnoses based on topology and morphology using the International Classification of Diseases Oncology version 3. For this study, we used the topology classification based on International Classification of Diseases (ICD) version 10. The cancer diagnoses were classified as all cancer types (‘all sites’) except non-melanoma skin cancer (ICD-10 codes C00–C96, excluding C44; also including D32, D33, D42, D43, D35.3, D35.4, D44.3, D44.4, D44.5, D45, D46, and D47), and seven major cancer sites, that is breast (female, C50), prostate (C61), skin melanoma (C43), colon (C18), rectal (C19–C20), lung (C33–C34), and kidney (C64). For Sweden, we did not present numbers of lung and kidney cancers separately due to known delays in reporting to the national cancer registry affecting year 2021.

Stage was coded according to tumor size (T), lymph node status (N), and distant metastases (M), applying the criteria in the Union for International Cancer Control (UICC) TNM classification of malignant tumors version 8 [11]. In Denmark, Iceland, Norway, and Sweden, pathological information was used if available, otherwise clinical information. Missing values of N (NX) and M (MX) were recorded as N0 and M0, which has been validated in Nordic data [12]. Information on TNM stage was incomplete for colon cancer (Iceland) and lung cancer (Iceland/Sweden), thus not presented. Furthermore, TNM stage was not available for Finland.

To define the populations at risk of cancer, we retrieved annual population counts by sex, year, age group (18–49, 50–69, and ≥70 years), and country from each National Statistics Office.

### Statistical methods

The data were prepared and aggregated into anonymous count data in each country by use of a common structured data call and analyzed in one central node (Sweden).

For each country, numbers of new cancer cases were counted annually, quarterly (Q1–Q4), and monthly during 2020/2021 and compared to the counts of the corresponding period averaged over 2017–2019 (pre-pandemic reference period). Country-specific crude incidence rates were calculated as annual number of cancer cases divided by number of individuals at risk by age group (18–49, 50–69 and ≥70 years), year, and sex, and reported as cases per 100,000 individuals at risk.

Incidence rate ratios (IRRs) with 95% confidence intervals (CIs) were estimated using Poisson regression with cancer counts as the outcome and individuals at risk as offset. The IRRs compared rates in 2020 and 2021 to the pre-pandemic period with adjustments for age and sex. The relative percentage change (PC) in incidence rate was derived from the incidence ratio as PC = [IRR – 1] * 100. Interactions between year and the following variables were also included: quarter (Q1, Q2, Q3, and Q4), month, age, and sex. Similarly, stage-specific incidence rates were modeled with each stage representing a separate outcome. We used a statistical significance level of *p* < 0.05. Analyses were performed with Stata 18.0/BE.

## Results

In the Nordic countries, 166,781 and 173,894 new cases of cancer were diagnosed in 2020 and 2021, respectively, compared to 173,047 reported cases in 2019 (Table 1, Supplemental Table S1). In Finland, Norway, and Sweden, the five most common cancer types were prostate, breast, lung, colon cancers, and skin melanoma, while in Denmark and Iceland, the order was breast, prostate, lung, colon cancers, and skin melanoma. For completeness, monthly numbers and crude incidence rates during 2020 and 2021 are presented in Supplemental Tables S2 and S3.

**Table 1:** Number of cancer cases in 2020 and 2021, and differences in numbers versus pre-pandemic period 2017–2019 per country, men and women aged 18+ years.

Patient group	Denmark		Finland		Iceland		Norway		Sweden	
	2020	2021	2020	2021	2020	2021	2020	2021	2020	2021
	*N* (diff^a^)	*N* (diff^b^)	*N* (diff^a^)	*N* (diff^b^)	*N* (diff^a^)	*N* (diff^b^)	*N* (diff^a^)	*N* (diff^b^)	*N* (diff^a^)	*N* (diff^b^)
**Total, annual, Q1–Q4**	39,987 (-216)	41,668 (1,465)	32,845 (-425)	33,804 (534)	1,765 (85)	1,847 (167)	34,208 (683)	35,332 (1,807)	57,976 (-3,078)	61,243 (189)
**Diagnosis period**										
Q1	9,872 (247)	9,516 (-109)	8,351 (0)	8,202 (-149)	413 (-18)	475 (44)	8,984 (312)	9,023 (351)	16,043 (-108)	15,357 (-794)
Q2	8,840 (-1,229)	10,283 (214)	7,568 (-924)	8,783 (291)	405 (-13)	439 (21)	7,930 (-716)	9,085 (439)	12,401 (-3,012)	15,974 (561)
Q3	10,136 (439)	10,261 (564)	8,097 (103)	8,126 (132)	429 (47)	441 (59)	8,159 (460)	8,279 (580)	13,878 (-35)	14,211 (298)
Q4	11,139 (326)	11,608 (795)	8,829 (396)	8,693 (260)	518 (69)	492 (43)	9,135 (627)	8,945 (437)	15,654 (76)	15,701 (123)
**Age, years**										
18–49	3,765 (-27)	3,741 (-51)	2,608 (-87)	2,675 (-20)	197 (-15)	218 (6)	3,527 (14)	3,362 (-151)	5,660 (-177)	5,641 (-196)
50–69	15,417 (-894)	15,867 (-444)	12,378 (-853)	12,524 (-707)	715 (-6)	732 (11)	13,027 (-386)	13,751 (338)	21,235 (-1,883)	22,028 (-1,090)
70+	20,805 (705)	22,060 (1,960)	17,859 (515)	18,605 (1,261)	853 (106)	897 (150)	17,654 (1,056)	18,219 (1,621)	31,081 (-1,019)	33,574 (1,474)
**Sex**										
Women	19,596 (-133)	20,348 (619)	15,663 (-364)	16,203 (176)	897 (36)	926 (65)	15,847 (146)	16,733 (1,032)	27,827 (-1,046)	29,503 (630)
Men	20,391 (-83)	21,320 (846)	17,182 (-61)	17,601 (358)	868 (49)	921 (102)	18,361 (538)	18,599 (776)	30,149 (-2,032)	31,740 (-441)
**Type of cancer**										
Breast (female)	4,892 (-123)	5,070 (55)	4,888 (-134)	5,107 (85)	281 (12)	287 (18)	3,822 (-158)	4,476 (496)	7,988 (-454)	8,973 (531)
Prostate	4,490 (-62)	4,644 (92)	5,051 (-201)	5,217 (-35)	242 (44)	269 (71)	5,102 (80)	5,230 (208)	9,056 (-1,777)	10,199 (-634)
Melanoma	2,715 (22)	2,921 (228)	1,569 (-182)	1,819 (68)	44 (-4)	51 (3)	2,670 (106)	2,766 (202)	4,603 (126)	5,034 (557)
Colon	3,197 (-285)	3,344 (-138)	2,345 (101)	2,428 (184)	119 (-34)	167 (14)	3,483 (54)	3,620 (191)	5,002 (91)	5,332 (421)
Rectum	1,350 (-215)	1,454 (-111)	1,299 (-4)	1,393 (90)	42 (-3)	57 (12)	1,300 (89)	1,216 (5)	2,065 (-90)	2,234 (79)
Lung	5,118 (189)	5,210 (281)	2,931 (11)	2,859 (-61)	190 (14)	170 (-6)	3,439 (22)	3,590 (173)	N/A	N/A
Kidney	1,048 (32)	1,122 (106)	991 (-25)	1,028 (12)	74 (16)	74 (16)	924 (-18)	955 (13)	N/A	N/A

a Difference in numbers 2020 to the average annual number 2017–2019.

b Difference in numbers 2021 to the average annual number 2017–2019.

### Changes in incidence rates by pandemic waves

**Figure 1:**
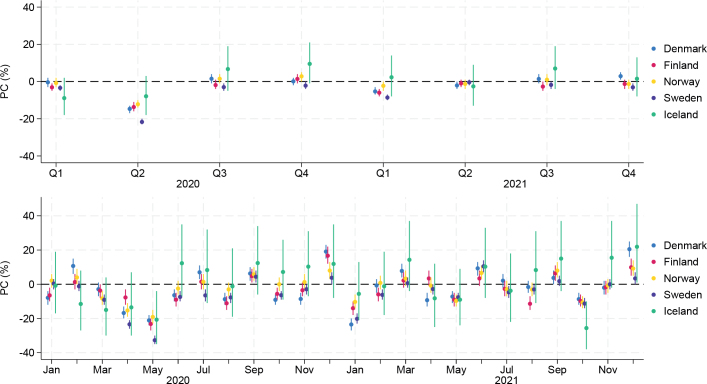
Adjusted^1^ percentage changes (PCs) in cancer incidence rates (all sites) comparing 2020 and 2021 versus 2017–2019 (top panel: quarterly, bottom panel: months). Estimates provided in Supplemental Table S4. ^1^Adjusted for age (18–49/50–69/70+) and sex (men/women).

During the first pandemic wave (Q2) in 2020, all countries experienced large declines in cancer incidence rates ranging from –21.7% (95% CI –23.3%; –20.2%) in Sweden to –7.9% (–17.7%; 3.0%) in Iceland compared to pre-pandemic rates (Figure 1, Supplemental Table S4). The declines were particularly pronounced in April and May 2020. During the remainder of 2020, rates were mostly in line with pre-pandemic rates, except for increased incidence rates in Denmark (19.1% [14.9%; 23.5%]), Finland (16.7% [11.8%; 21.9%]), Norway (8.0% [3.5%; 12.8%]), and Sweden (3.8% [0.4%; 7.2%]) during December. In Iceland, the incidence rates were substantially increased during both Q3 (6.7% [–4.5%; 19.2%]) and Q4 (9.5% [–1.0%; 21.2%]) 2020.

During 2021, the largest declines were observed in January (Denmark: –23.5% [95% CI –26.7%; –20.1%], Finland: –14.0% [–17.7%; –10.2%], and Sweden: –20.2% [–22.7%; –17.5%]), although Norway also experienced substantial declines (–10.3% [–13.9%; –6.5%]), while the decline in Iceland was –5.6% (–21.4%; 13.4%) (Figure 1). By the end of 2021, Sweden experienced a decline in cancer incidence in Q4 (–3.1% [–4.8%; –1.3%]). Yet, all countries showed the same pattern of declines in October 2021 and increased rates in December that same year.

**Figure 2:**
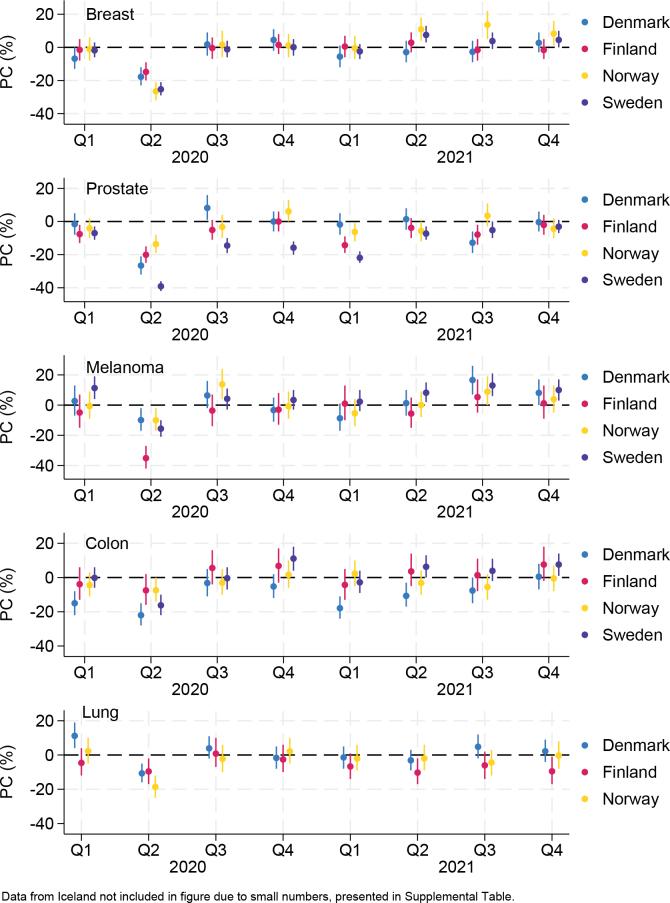
Adjusted percentage changes (PCs) in cancer incidence rates comparing 2020 and 2021 versus 2017–2019, by cancer site. Estimates provided in Supplemental Table S5.

Breast and prostate cancer incidence rates declined the most during Q2 2020 in all countries, with the largest reduction in prostate cancer rates (–39.1% [95% CI –42.2%; –35.9%]) in Sweden (Figure 2, Supplemental Table S5). In Norway, breast cancer rates were elevated toward the end of 2021, while there was no substantial catch-up in Denmark, Finland, and Sweden. Prostate cancer rates in Sweden continued to be lower than pre-pandemic rates during most of 2020 and 2021, while rates were lower in Finland and Norway during the second pandemic wave (Q1, 2021). Rates of skin melanoma were consistently decreased in all countries during the first pandemic wave (Q2, 2020), with some indication of elevated rates during Q3 and Q4 2021. Colon cancer rates were decreased in Denmark and Sweden during Q2 2020 and were also lower than pre-pandemic rates in Denmark during Q1 and Q2 2021. In Finland and Sweden, there was some indication of elevated colon cancer rates in 2021. Lung cancer rates were decreased compared to pre-pandemic rates in Denmark, Finland, and Norway during the first pandemic wave and later also decreased in Finland during Q4 2021.

### Changes in annual incidence rates

**Figure 3:**
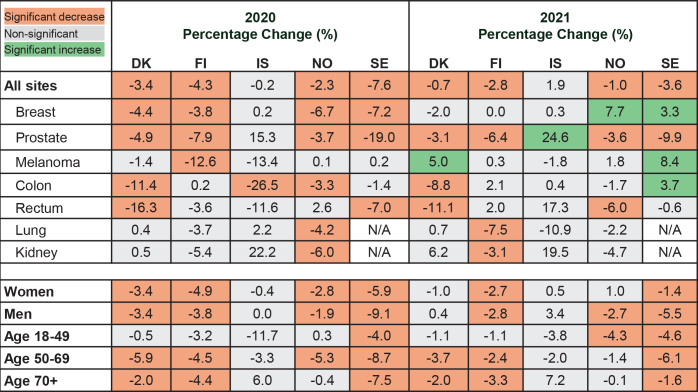
Adjusted percentage changes (PCs) of annual cancer incidence rates comparing 2020 and 2021 versus 2017–2019, by cancer site, sex and age. Estimates provided in Supplemental Table S4. Countries are Denmark (DK), Finland (FI), Iceland (IS), Norway (NO) and Sweden (SE).

During 2020, the largest decline in annual cancer incidence occurred in Sweden (–7.6% [95% CI –8.5%; –6.8%]), followed by Finland (–4.3% [–5.5%; –3.1%]), Denmark (–3.4% [–4.5%; –2.3%]), and Norway (–2.3% [–3.5%; –1.1%]) (Figure 3, Supplemental Table S4). Reductions in annual breast cancer incidence rates ranged from –7.2% (–9.5%; –4.8%) in Sweden to –3.8% (–6.9%; –0.7%) in Finland, with no reduction in Iceland, while the annual prostate cancer rates declined significantly in Sweden (–19.0% [–20.8%; –17.1%]), Finland (–7.9% [–10.8%; –4.9%]), Denmark (–4.9% [–8.0%; –1.6%]), and Norway (–3.7% [–6.7%; –0.6%]). For other cancer sites, the patterns varied across countries with the annual skin melanoma rate declining in Finland (–12.6% [–17.4%; –7.6%]), colon cancer rates declining in Denmark (–11.4% [–14.9%; –7.8%]) and Iceland (–26.5% [–39.9%; –10.1%]), rectal cancer in Denmark (–16.3% [–21.2%; –11.1%]), and lung (–4.2% [–7.8%; –0.4%]) and kidney cancers (–6.0% [–12.7%; 1.3%]) in Norway. Reductions in annual cancer incidence rates of all sites were mainly observed in individuals aged 50–69 years, ranging from –8.7% (–10.1%; –7.3%) in Sweden to –4.5% (–6.4%; –2.5%) in Finland, and a decline of –3.3% (–11.2%; 5.2%) in Iceland. Among individuals aged ≥70 years, annual reductions were present in Sweden (–7.5% [–8.6%; –6.3%]), Finland (–4.4% [–6.1%; –2.8%]), and Denmark (–2.0% [–3.5%; –0.4%]), and in Sweden, also among those aged 18–49 years (–4.0% [–6.8%; –1.1%]).

During 2021, substantial annual reductions in cancer incidence (all sites) remained compared to the pre-pandemic period in Sweden (–3.6% [95% CI –4.4%; –2.7%]) and Finland (–2.8% [–3.9%; –1.5%]), while moderate declines occurred in Denmark (–0.7% [–1.8%; 0.4%]) and Norway (–1.0% [–2.2%; 0.2%]) (Figure 3). However, the annual breast cancer incidence rates increased significantly in Norway (7.7% [4.1%; 11.5%]) and Sweden (3.3% [0.8%; 5.8%]) compared to 2017–2019. In contrast, the annual prostate cancer incidence rates decreased in Finland (–6.4% [–9.3%; –3.4%]), Norway (–3.6% [–6.6%; –0.5%]), Sweden (–9.9% [–11.9%; –7.9%]), and Denmark (–3.1% [–6.3%; 0.1%]), while it increased in Iceland (24.6% [7.9%; 43.9%]). In Sweden, the incidence rates of skin melanoma and colon cancer increased during 2021 compared to the pre-pandemic period, while skin melanoma rates increased, and colon cancer rates decreased in Denmark. In 2021, annual cancer incidence declined among individuals 50–69 years in Denmark, Sweden, and Finland, but not in Norway and Iceland. Significant declines were also found in ages 18–49 years in Norway and Sweden, and among the older age group (≥70 years) in Finland and Sweden. Both men and women experienced reductions in cancer incidence in all countries, except in Iceland, during 2020. During 2021, cancer incidence rates declined moderately in men and women in Sweden and Finland, while only among men in Norway.

### Changes in stage-specific annual incidence rates

**Figure 4:**
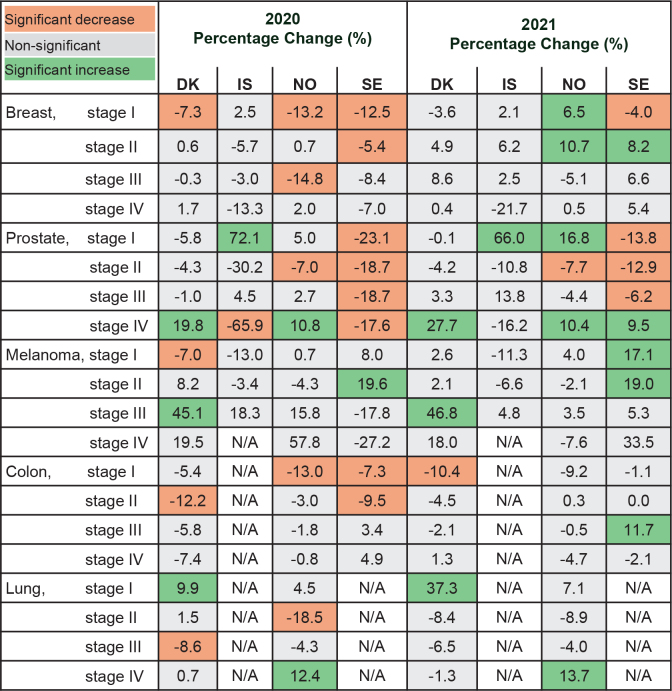
Adjusted percentage changes (PCs) of annual stage-specific incidence rates comparing 2020 and 2021 versus 2017–2019, by cancer site. Estimates provided in Supplemental Table S6. Countries are Denmark (DK), Iceland (IS), Norway (NO) and Sweden (SE).

Compared to the pre-pandemic period, the reductions in stage I breast cancer incidence were particularly large during 2020 in Denmark (–7.3% [95% CI –11.7%; –2.7%]), Norway (–13.2% [–17.8%; –8.4%]), and Sweden (–12.5% [–15.6%; –9.3%]) (Figure 4; Supplemental Table S6). In 2021, there was an increase in both stage I and stage II breast cancer rates in Norway (6.5% [1.3%; 11.9%] and 10.7% [4.6%; 17.1%], respectively), and in stage II breast cancer in Denmark (4.9% [–0.6%; 10.6%]) and Sweden (8.2% [4.0%; 12.5%]). However, in Sweden, stage I breast cancer incidence continued to decline (–4.0% [–7.3%; –0.7%]) in 2021. For prostate cancer, incidence rates substantially declined across stages I–IV in Sweden during 2020, and stage I and II rates also declined in 2021, while stage IV rates increased. In Iceland and Norway, stage I prostate cancer rates increased in both 2020 and 2021, while in Denmark and Norway, stage IV rates increased in both years. For malignant melanoma, stages I and II malignant melanoma rates increased in both 2020 and 2021 in Sweden, while stage I melanoma rates increased in 2021 in Norway. For colon cancer, stage I incidence rates declined during 2020 in Norway, while stage II rates declined in Denmark and Sweden during 2020. During 2021, moderate increases in stage III rates were observed in Sweden. For lung cancer, stage I incidence rates increased in Denmark during both 2020 and 2021, while in Norway, the patterns were less clear with declines in stage II lung cancer incidence during 2020, followed by increasing rates of stage I in 2021 and stage IV in both years.

## Discussion

In this large population-based study, we found substantial declines in cancer incidence rates across the Nordic countries in 2020, especially during the first pandemic wave. Importantly, we also found moderate declines in the annual cancer incidence rates in 2021, particularly in Sweden and Finland, and significant declines during the second pandemic wave (Q1, 2021) in Denmark, Finland, Norway, and Sweden. During the third wave at the end of 2021, the cancer incidence rates declined in October but increased in December across all countries.

The early reductions in cancer incidence during 2020 were particularly pronounced for female breast cancer. Specifically, women of breast cancer screening ages experienced the largest reductions in incidence, which was reflected in lower rates of stage I breast cancer in Denmark, Norway, and Sweden, and stage II breast cancer in Sweden. Prostate cancer rates declined substantially in Sweden, Finland, Denmark, and Norway during 2020, with Finland, Norway, and Sweden exhibiting a lower annual rate also in 2021 compared to the pre-pandemic period. For melanoma, colon, rectum, lung, and kidney cancers, the changes in incidence rates were less consistent across countries, yet with substantial declines during the first pandemic wave. Although screening ages were mostly affected, significant reductions in annual cancer incidence rates were also found among younger (<50 years) and older (≥70 years) individuals in both 2020 and 2021, which may reflect a generally lower healthcare utilization. The vaccination roll-out during 2021 may have partly mitigated the impact of the third pandemic wave. Yet, except for breast cancer, there was limited evidence of a catch-up in 2021 of the substantial declines observed in the early phase of the pandemic in 2020.

The underlying reasons for the reductions in cancer incidence during the first 2 years of the pandemic likely include reduced screening activities, changed healthcare seeking behavior, and reduced healthcare capacity to conduct timely diagnostics during periods of pandemic waves. Screening programs for breast, colorectal, and cervical cancer were temporarily halted (fully or partly) in Norway, Sweden, and Iceland during the early phase of the pandemic in 2020. After screening activities were resumed, lower attendance rates remained [6]. In Norway, the breast cancer screening programme resumed during the end of Summer 2020, yet at a lower capacity, while in Sweden, delays were present throughout 2020 [6]. In Norway, a catch-up in breast cancer incidence, especially of stages I and II cancers, was observed in 2021, likely due to increased activity in the screening programme to reduce the backlog. Lower rates of screen-detected breast cancer during 2020 have been reported in both Norway and Sweden [13, 14]. Breast cancer screening in Denmark was not halted during the COVID19 pandemic, but the screening activity dropped with the onset of the initial lockdown and returned to near pre-pandemic levels by the end of 2020. However, lower participation was observed among immigrants and women with low-income background [15]. In Finland, a few regions halted breast cancer screening temporarily for a short period, yet lower attendance was reported.

In Iceland, early implementation of SARS-CoV-2 testing, case tracing, and quarantining in combination with strict entry control at the only major international gateway to the country meant that community spread remained low, and that cancer diagnostic activity remained stable during the pandemic, except for a 2-month suspension of breast and cervical cancer screening during the first pandemic wave from March to May 2020 [16]. This resulted in fewer cases diagnosed during the first wave, which was followed by a catch-up during the remainder of 2020, yielding the same annual incidence in 2020, as in previous years [17].

In Denmark, colorectal cancer screening operations continued during the pandemic, yet lower rates of screen-detected colorectal cancer cases were observed [18]. However, the introduction of the Danish colorectal cancer screening programme in 2014 led to a sharp increase in both colon and rectum cancer rates in the following years before returning to a normal level around 2019–2020. Consequently, the cancer rates in the comparison period 2017–2019 for these two cancers may be higher than those in 2020 and 2021 due to the introduction of the screening, rather than reflecting a pandemic effect.

Prostate cancer rates declined substantially in all Nordic countries, except Iceland, both during 2020 and 2021. In Sweden, lower rates of Prostate Specific Antigen (PSA) testing in non-symptomatic men were reported, which could explain the lower prostate cancer rates [19]. However, of note is that the introduction of Magnetic Resonance Imaging (MRI) in men with high PSA in 2019 may also have affected the incidence rates in the following years. In Denmark, Norway, and Sweden, stage IV prostate cancer incidence increased in 2021, which remains unexplained.

Throughout 2020/2021, healthcare services remained open to individuals with non-COVID-19-related diseases. However, several reports indicated fewer visits to healthcare services in 2020 [20–22]. A reduction in the number of pathology reports during 2020 also indicates that fewer individuals sought healthcare for cancer-related symptoms [6]. During 2021, the nationwide vaccination programmes were rolled out in all Nordic countries and likely influenced the healthcare-seeking patterns. Thus, despite continued community transmission of SARS-CoV-2 in the third pandemic wave, increases in cancer incidence rates were observed at the end of 2021. However, in Denmark, increases at the end of the year (and corresponding decreases at the start of the year) have been noted both before and after the pandemic. This pattern is likely related to the implementation of a new registration system for the National Patient Register in 2019.

Our findings are in line with several international studies, which have reported lower cancer rates during the early phases of the pandemic in the pre-vaccination period [2, 3]. Among studies that assessed the impact of the second and third pandemic waves in 2021, most have reported incidence rates similar to the pre-pandemic years, with no substantial catch-up in 2021 of the deficit from the first pandemic wave [23–26]. Studies from the UK reported declining incidence rates in 2020, while rates during 2021 were similar to the pre-pandemic rates for most cancer types, with one exception being prostate cancer [24, 26]. A Canadian study also reported similar rates in 2021 as the pre-pandemic period with no catch-up of previous deficits across a wide range of cancer sites [23]. In contrast, a German study showed decreased cancer incidence rates also during the second pandemic wave, although not for breast cancer [25]. Our finding of a catch-up in breast cancer rates in Norway has been reported previously, also in comparison to Dutch data, where a similar catch-up was not found [13, 27]. Yet, our finding of lower rates of stage I breast cancer in the initial phase of the pandemic is consistent with other reports [13, 28–32]. Similarly, rates of early-stage colorectal cancer declined in several other countries during 2020 [28–31, 33–35], as did the rates of prostate cancer in general and early-stage disease in particular [28–31, 36].

In this large international comparison of cancer incidence rates during the first 2 years of the COVID-19 pandemic, we used quality-assured and essentially complete information on incident cancer cases in a region with 27 million residents. The Nordic countries have mature cancer registries embedded within nationwide tax-funded healthcare systems, which also provide organized cancer screening programs [8, 37]. The classification of diagnoses and TNM staging follows the same coding systems in all Nordic countries, which facilitated the use of a structured data call to all countries to ensure similar inclusions and definitions for the analyzed data.

A limitation of the study was the ecological study design, which only provided descriptive cancer incidence trends. Thus, the impact of the pandemic development and mitigation efforts, such as lockdowns, travel restrictions, and vaccination programs, cannot be assessed directly at the individual level, but only inferred indirectly from the timing of these efforts during different periods of 2020 and 2021. Adjustments for age and sex ensured that the underlying annual increases in cancer incidence rates of around 1.2–2.2% due to demographic changes were accounted for [10]. However, due to the broad age groups, residual confounding by age cannot be excluded. Another limitation was that the comparison period was combined into an average of 2017–2019 without adjustments for population changes. Additionally, information on stage was unavailable for Finland, and some comparisons for Iceland were hampered by limited statistical precision.

In conclusion, we found large declines in cancer incidence rates in the Nordic countries during the first 2 years of the COVID-19 pandemic, especially during the first and second pandemic waves. The reductions were particularly pronounced in Sweden, yet were also substantial in the other countries although less so for Iceland, which implemented stringent SARS-CoV-2 testing and pandemic containment strategies early. The reduction in screening activities is likely the main explanation for the declines in female breast cancer incidence and changes in stage distribution, while the reduction in prostate cancer incidence may be related to less PSA testing in primary care. However, our results suggest that diagnostic delay may persist for certain cancer types, which emphasizes the importance of continued monitoring of the potential long-term consequences of the COVID-19 pandemic on cancer stage distribution and survival.

## Supplementary Material

Changes in cancer incidence and stage during the COVID-19 pandemic in 2020–2021 in the Nordic countries

## Data Availability

This study was based on national cancer registry data. The data are available from the registry holder of each specific health registry under the use of appropriate ethical and legal permissions, including the General Data Protection Regulation (GDPR). Further details and other data that support the findings of this study are available from the corresponding author upon request.
