# Supplementary material for: Changes in cancer incidence and stage during the COVID-19 pandemic in 2020–2021 in the Nordic countries
Source: Acta Oncol. 2025 Feb 12;64:42079. doi: 10.2340/1651-226X.2025.42079 (PMC11833327; doi:10.2340/1651-226X.2025.42079)

Supplementary material has been published as submitted. It has not been copyedited, or typeset by Acta Oncologica

SUPPLEMENTAL MATERIAL

Supplemental Table S1. Number of cancer cases (all sites) for diagnosis years 2017-2021 in the Nordic countries, men and women aged 18+ years.

Supplemental Table S2. Number of cancer cases per month 2020 and 2021, and differences in numbers versus pre-pandemic period 2017-2019 per country.

Supplemental Table S3. Incidence rates per 100,000 (crude) of cancer in 2017-2019 (annual average), 2020 and 2021 per country, men and women aged 18+ years.

Supplemental Table S4. Age and sex-adjusted incidence rate ratios (IRR) comparing 2020 and 2021 versus 2017-2019, by diagnostic period (annual, quarterly, monthly), age, sex and cancer site per country. Corresponding to Figure 1 and 3.

Supplemental Table S5. Age and sex-adjusted incidence rate ratios (IRR) comparing 2020 and 2021 versus 2017-2019, by diagnostic period (quarterly) and cancer site per country. Corresponding to Figure 2.

Supplemental Table S6. Age and sex-adjusted stage-specific incidence rate ratios (IRR) of annual rates 2020 and 2021 vs pre-pandemic 2017-2019 per country. Corresponding to Figure 4.

Supplemental Figure S1. Weekly incidence (N) of COVID-19 per country 2020-2021.

Supplemental Table S1. Number of cancer cases (all sites) for diagnosis years 2017-2021 in the Nordic countries, men and women aged 18+ years.

	2017<br>N	2018<br>N	2019<br>N	2020<br>N	2021<br>N
Denmark	39822	39798	40989	39987	41668
Finland	32961	33061	33787	32845	33804
Iceland	1625	1675	1740	1765	1847
Norway	33006	33549	34019	34208	35332
Sweden	59769	60882	62512	57976	61243
**Total**	**167183**	**168965**	**173047**	**166781**	**173894**

Supplemental Table S2. Number of cancer cases per month 2020 and 2021, and differences in numbers versus pre-pandemic period 2017-2019 per country.

	DENMARK		FINLAND		ICELAND		NORWAY		SWEDEN	
	2020 N (diff[a])	2021 N (diff[b])	2020 N (diff[a])	2021 N (diff[b])	2020 N (diff[a])	2021 N (diff[b])	2020 N (diff[a])	2021 N (diff[b])	2020 N (diff[a])	2021 N (diff[b])
**Total (annual)**	39987 (-216)	41668 (1465)	32845 (-425)	33804 (534)	1765 (85)	1847 (167)	34208 (683)	35332 (1807)	57976 (-3078)	61243 (189)
**Diagnosis period**										
Jan	3042 (-165)	2561 (-646)	2821 (-101)	2624 (-298)	157 (7)	153 (3)	3290 (204)	2949 (-137)	5657 (173)	4554 (-930)
Feb	3388 (415)	3081 (108)	2710 (119)	2548 (-43)	132 (-10)	151 (9)	2920 (232)	2886 (198)	5199 (82)	4994 (-123)
Mar	3442 (-2)	3874 (430)	2820 (-18)	3030 (192)	124 (-15)	171 (32)	2774 (-123)	3188 (291)	5187 (-363)	5809 (259)
Apr	2610 (-438)	2884 (-164)	2519 (-126)	2856 (211)	116 (-11)	126 (-1)	2332 (-303)	2783 (148)	3946 (-1068)	5068 (54)
May	2880 (-665)	3432 (-113)	2393 (-626)	2870 (-149)	131 (-26)	154 (-3)	2535 (-467)	2885 (-117)	3840 (-1712)	5336 (-216)
Jun	3350 (-127)	3967 (490)	2656 (-173)	3057 (228)	158 (24)	159 (25)	3063 (54)	3417 (408)	4615 (-232)	5570 (723)
Jul	3473 (319)	3360 (206)	2513 (114)	2439 (40)	133 (16)	121 (4)	2413 (134)	2357 (78)	4094 (-166)	4223 (-37)
Aug	3024 (-193)	3303 (86)	2629 (-235)	2652 (-212)	130 (5)	146 (21)	2664 (33)	2714 (83)	4410 (-238)	4688 (40)
Sep	3639 (313)	3598 (272)	2955 (224)	3035 (304)	166 (26)	174 (34)	3082 (293)	3208 (419)	5374 (369)	5300 (295)
Oct	3417 (-233)	3477 (-173)	2974 (-77)	2877 (-174)	194 (22)	138 (-34)	3136 (132)	2860 (-144)	5445 (-216)	5225 (-436)
Nov	3511 (-218)	3814 (85)	2992 (-13)	3087 (82)	178 (25)	191 (38)	3177 (175)	3176 (174)	5378 (-10)	5606 (218)
Dec	4211 (777)	4317 (883)	2863 (487)	2729 (353)	146 (22)	163 (39)	2822 (320)	2909 (407)	4831 (302)	4870 (341)

[a] Difference in numbers 2020 to the average annual number 2017-2019.
[b] Difference in numbers 2021 to the average annual number 2017-2019.

Supplemental Table S3. Incidence rates per 100,000 (crude) of cancer in 2017-2019 (annual average), 2020 and 2021 per country, men and women aged 18+ years.

	DENMARK			FINLAND			ICELAND		
	**2017-2019**	**2020**	**2021**	**2017-2019**	**2020**	**2021**	**2017-2019**	**2020**	**2021**
	Rate*	Rate*	Rate*	Rate*	Rate*	Rate*	Rate*	Rate*	Rate*
**Total, annual, Q1-Q4**	868.9 [864.0-873.8]	855.8 [847.5-864.3]	886.8 [878.3-895.3]	747.0 [742.4-751.7]	732.5 [724.6-740.4]	750.8 [742.8-758.8]	618.4 [601.6-635.7]	620.2 [592.0-649.9]	638.0 [609.6-667.8]
**Diagnosis period**									
Q1	832.0 [822.5-841.7]	845.2 [828.6-862.0]	810.1 [794.0-826.5]	750.0 [740.8-759.4]	744.9 [729.1-761.1]	728.7 [713.1-744.6]	634.1 [600.4-669.6]	580.5 [527.2-639.3]	656.3 [599.9-718.1]
Q2	870.5 [860.7-880.3]	756.8 [741.2-772.7]	875.4 [858.6-892.5]	762.8 [753.4-772.2]	675.1 [660.0-690.5]	780.3 [764.1-796.8]	615.4 [582.3-650.5]	569.3 [516.5-627.5]	606.6 [552.4-666.1]
Q3	838.3 [828.7-848.0]	867.8 [851.0-884.8]	873.5 [856.8-890.6]	718.0 [709.0-727.1]	722.3 [706.7-738.2]	721.9 [706.4-737.8]	562.4 [530.8-596.0]	603.0 [548.6-662.9]	609.3 [555.0-669.0]
Q4	934.7 [924.6-945.0]	953.6 [936.1-971.5]	988.2 [970.4-1006.3]	757.4 [748.1-766.8]	787.6 [771.3-804.2]	772.3 [756.2-788.7]	661.6 [627.2-697.9]	728.1 [668.1-793.6]	679.8 [622.3-742.6]
**Age, years**									
18-49	160.4 [157.5-163.4]	159.6 [154.6-164.8]	158.6 [153.6-163.8]	124.6 [121.9-127.3]	120.6 [116.1-125.3]	123.3 [118.7-128.0]	133.8 [123.8-144.6]	118.2 [102.8-135.9]	128.7 [112.7-147.0]
50-69	1117.9 [1108.0-1127.8]	1052.2 [1035.7-1069.0]	1076.9 [1060.3-1093.8]	903.6 [894.8-912.6]	863.4 [848.3-878.7]	882.4 [867.1-898.0]	905.5 [868.1-944.5]	875.4 [813.5-942.0]	887.5 [825.5-954.2]
70+	2500.1 [2480.2-2520.1]	2451.6 [2418.5-2485.2]	2543.8 [2510.4-2577.6]	2101.0 [2083.0-2119.1]	2010.8 [1981.6-2040.5]	2036.9 [2007.8-2066.3]	2222.5 [2132.3-2316.4]	2355.2 [2202.3-2518.7]	2381.6 [2230.8-2542.7]
**Sex**									
Women	842.5 [835.7-849.3]	828.2 [816.7-839.9]	855.4 [843.7-867.2]	704.3 [698.0-710.6]	684.7 [674.0-695.5]	706.0 [695.2-717.0]	646.9 [622.4-672.3]	647.2 [606.2-691.0]	656.1 [615.2-699.8]
Men	895.9 [888.9-903.0]	884.2 [872.1-896.4]	919.0 [906.7-931.4]	791.7 [784.9-798.6]	782.2 [770.6-794.0]	797.3 [785.6-809.2]	591.0 [568.1-614.9]	594.6 [556.4-635.5]	620.8 [582.0-662.2]
**Type of cancer**									
Breast (female)	214.2 [210.8-217.6]	206.8 [201.0-212.6]	213.1 [207.3-219.1]	220.7 [217.2-224.2]	213.7 [207.8-219.7]	222.5 [216.5-228.7]	202.2 [188.7-216.6]	202.8 [180.4-227.9]	203.4 [181.1-228.3]
Prostate	199.2 [195.9-202.6]	194.7 [189.1-200.5]	200.2 [194.5-206.0]	241.2 [237.4-245.0]	229.9 [223.7-236.4]	236.3 [230.0-242.8]	142.8 [131.8-154.8]	165.8 [146.2-188.0]	181.3 [160.9-204.3]
Melanoma	58.2 [57.0-59.5]	58.1 [56.0-60.3]	62.2 [60.0-64.5]	39.3 [38.3-40.4]	35.0 [33.3-36.8]	40.4 [38.6-42.3]	17.8 [15.1-20.9]	15.5 [11.5-20.8]	17.6 [13.4-23.2]
Colon	75.3 [73.8-76.7]	68.4 [66.1-70.8]	71.2 [68.8-73.6]	50.4 [49.2-51.6]	52.3 [50.2-54.5]	53.9 [51.8-56.1]	56.3 [51.4-61.7]	41.8 [34.9-50.0]	57.7 [49.6-67.1]
Rectum	33.8 [32.9-34.8]	28.9 [27.4-30.5]	30.9 [29.4-32.6]	29.3 [28.4-30.2]	29.0 [27.4-30.6]	30.9 [29.4-32.6]	16.7 [14.1-19.7]	14.8 [10.9-20.0]	19.7 [15.2-25.5]
Lung	106.5 [104.8-108.3]	109.5 [106.6-112.6]	110.9 [107.9-113.9]	65.6 [64.2-67.0]	65.4 [63.0-67.8]	63.5 [61.2-65.9]	64.9 [59.6-70.7]	66.8 [57.9-77.0]	58.7 [50.5-68.2]
Kidney	22.0 [21.2-22.8]	22.4 [21.1-23.8]	23.9 [22.5-25.3]	22.8 [22.0-23.6]	22.1 [20.8-23.5]	22.8 [21.5-24.3]	21.2 [18.3-24.6]	26.0 [20.7-32.7]	25.6 [20.4-32.1]

	NORWAY			SWEDEN		
	**2017-2019**	**2020**	**2021**	**2017-2019**	**2020**	**2021**
	Rate*	Rate*	Rate*	Rate*	Rate*	Rate*
**Total, annual, Q1-Q4**	800.7 [795.8-805.7]	802.2 [793.7-810.7]	822.0 [813.5-830.6]	755.6 [752.1-759.0]	706.6 [700.8-712.3]	741.2 [735.3-747.0]
**Diagnosis period**						
Q1	828.5 [818.5-838.6]	842.7 [825.5-860.3]	839.7 [822.5-857.2]	799.5 [792.4-806.6]	782.1 [770.1-794.3]	743.4 [731.7-755.2]
Q2	826.1 [816.1-836.2]	743.8 [727.7-760.4]	845.5 [828.3-863.0]	762.9 [756.0-769.9]	604.5 [594.0-615.3]	773.3 [761.4-785.3]
Q3	735.5 [726.1-745.1]	765.3 [748.9-782.1]	770.5 [754.0-787.2]	688.7 [682.1-695.3]	676.5 [665.4-687.9]	687.9 [676.7-699.3]
Q4	812.8 [802.9-822.8]	856.9 [839.5-874.6]	832.4 [815.4-849.9]	771.1 [764.2-778.2]	763.1 [751.2-775.1]	760.0 [748.2-772.0]
**Age, years**						
18-49	153.4 [150.5-156.4]	153.8 [148.8-159.0]	153.4 [150.5-156.4]	139.6 [137.6-141.7]	134.1 [130.7-137.7]	133.2 [129.8-136.8]
50-69	1063.1 [1052.7-1073.5]	1006.3 [989.2-1023.8]	1063.1 [1052.7-1073.5]	962.2 [955.0-969.4]	878.0 [866.3-889.9]	903.7 [891.8-915.7]
70+	2615.1 [2592.3-2638.2]	2608.8 [,2570.6-2647.6]	2615.1 [2592.3-2638.2]	2142.4 [2128.9-2155.9]	1983.5 [1961.6-2005.7]	2109.0 [2086.6-2131.7]
**Sex**						
Women	752.2 [745.5-759.0]	746.2 [734.6-757.9]	752.2 [745.5-759.0]	713.6 [708.9-718.4]	678.5 [670.6-686.5]	714.5 [706.4-722.7]
Men	848.9 [841.8-856.2]	857.8 [845.5-870.3]	848.9 [841.8-856.2]	797.6 [792.6-802.7]	734.6 [726.4-743.0]	767.8 [759.4-776.2]
**Type of cancer**						
Breast (female)	190.7 [187.3-194.1]	180.0 [174.3-185.8]	190.7 [187.3-194.1]	208.6 [206.1-211.2]	194.8 [190.5-199.1]	217.3 [212.9-221.9]
Prostate	239.2 [235.4-243.0]	238.3 [231.9-245.0]	239.2 [235.4-243.0]	268.5 [265.6-271.4]	220.7 [216.2-225.3]	246.7 [242.0-251.5]
Melanoma	61.2 [59.9-62.6]	62.6 [60.3-65.0]	61.2 [59.9-62.6]	55.4 [54.5-56.3]	56.1 [54.5-57.7]	60.9 [59.3-62.6]
Colon	81.9 [80.3-83.5]	81.7 [79.0-84.4]	81.9 [80.3-83.5]	60.8 [59.8-61.8]	61.0 [59.3-62.7]	64.5 [62.8-66.3]
Rectum	28.9 [28.0-29.9]	30.5 [28.9-32.2]	28.9 [28.0-29.9]	26.7 [26.0-27.3]	25.2 [24.1-26.3]	27.0 [25.9-28.2]
Lung	81.6 [80.0-83.2]	80.6 [78.0-83.4]	81.6 [80.0-83.2]	N/A	N/A	N/A
Kidney	22.5 [21.7-23.3]	21.7 [20.3-23.1]	22.5 [21.7-23.3]	N/A	N/A	N/A

*Rate per 100,000. Annual average for 2017-2019.

Supplemental Table S4. Age and sex-adjusted incidence rate ratios (IRR) comparing 2020 and 2021 versus 2017-2019, by diagnostic period (annual, quarterly, monthly), age, sex and cancer site per country. Corresponding to Figure 1 and 3.

	DENMARK				FINLAND				ICELAND			
	**2020**		**2021**		**2020**		**2021**		**2020**		**2021**	
	**IRR (95% CI)**	**PC %**	**IRR (95% CI)**	**PC %**	**IRR (95% CI)**	**PC %**	**IRR (95% CI)**	**PC %**	**IRR (95% CI)**	**PC %**	**IRR (95% CI)**	**PC %**
**Total (annual),***	0.97 [0.96-0.98]	-3.4	0.99 [0.98-1.00]	-0.7	0.96 [0.94-0.97]	-4.3	0.97 [0.96-0.98]	-2.8	1.00 [0.95-1.05]	-0.2	1.02 [0.97-1.08]	1.9
**Diagnosis period (quarterly)***												
Q1: Jan-Mar	1.00 [0.97-1.02]	-0.4	0.95 [0.93-0.97]	-5.3	0.97 [0.95-0.99]	-3.1	0.94 [0.92-0.96]	-6.0	0.91 [0.82-1.02]	-8.9	1.02 [0.92-1.14]	2.3
Q2: Apr-Jun	0.85 [0.83-0.87]	-14.7	0.98 [0.96-1.00]	-2.1	0.86 [0.84-0.89]	-13.7	0.99 [0.97-1.01]	-1.0	0.92 [0.82-1.03]	-7.9	0.97 [0.87-1.09]	-2.6
Q3: Jul-Sep	1.02 [0.99-1.04]	1.5	1.01 [0.99-1.04]	1.4	0.98 [0.96-1.01]	-1.9	0.97 [0.95-1.00]	-2.7	1.07 [0.95-1.19]	6.7	1.07 [0.96-1.19]	7.0
Q4: Oct-Dec	1.00 [0.98-1.02]	0.1	1.03 [1.01-1.05]	2.9	1.01 [0.99-1.04]	1.4	0.99 [0.96-1.01]	-1.3	1.10 [0.99-1.21]	9.5	1.02 [0.92-1.13]	1.5
**Diagnosis period (monthly)***												
Jan	0.92 [0.88-0.96]	-7.9	0.77 [0.73-0.80]	-23.5	0.94 [0.90-0.98]	-6.5	0.86 [0.82-0.90]	-14.0	0.99 [0.83-1.19]	-0.8	0.94 [0.79-1.13]	-5.6
Feb	1.11 [1.06-1.15]	10.7	0.99 [0.95-1.03]	-0.7	1.01 [0.97-1.06]	1.3	0.94 [0.90-0.98]	-5.9	0.89 [0.73-1.08]	-11.5	0.99 [0.82-1.19]	-1.2
Mar	0.97 [0.93-1.01]	-2.9	1.08 [1.04-1.12]	7.8	0.96 [0.92-1.00]	-3.7	1.02 [0.98-1.07]	2.2	0.85 [0.70-1.04]	-15.0	1.14 [0.96-1.37]	14.3
Apr	0.83 [0.80-0.87]	-16.8	0.91 [0.87-0.95]	-9.3	0.92 [0.88-0.97]	-7.7	1.03 [0.99-1.08]	3.4	0.87 [0.70-1.07]	-13.5	0.92 [0.75-1.12]	-8.2
May	0.79 [0.76-0.82]	-21.1	0.93 [0.89-0.96]	-7.2	0.77 [0.73-0.80]	-23.2	0.91 [0.87-0.95]	-9.0	0.79 [0.65-0.96]	-20.7	0.91 [0.76-1.09]	-9.0
Jun	0.94 [0.90-0.97]	-6.4	1.09 [1.05-1.13]	9.3	0.91 [0.87-0.95]	-9.0	1.03 [0.99-1.08]	3.4	1.12 [0.93-1.35]	12.3	1.10 [0.92-1.33]	10.3
Jul	1.07 [1.03-1.11]	7.0	1.02 [0.98-1.06]	2.1	1.01 [0.97-1.06]	1.5	0.97 [0.93-1.02]	-2.7	1.08 [0.89-1.32]	8.3	0.96 [0.78-1.18]	-3.8
Aug	0.91 [0.88-0.95]	-8.7	0.98 [0.95-1.02]	-1.6	0.89 [0.85-0.93]	-11.1	0.89 [0.85-0.93]	-11.4	0.99 [0.81-1.21]	-1.2	1.08 [0.89-1.31]	8.3
Sep	1.06 [1.02-1.10]	6.3	1.04 [1.00-1.08]	3.7	1.05 [1.01-1.09]	4.8	1.06 [1.02-1.11]	6.4	1.12 [0.94-1.34]	12.4	1.15 [0.96-1.37]	15.0
Oct	0.91 [0.88-0.94]	-9.1	0.91 [0.88-0.95]	-8.7	0.94 [0.91-0.98]	-5.6	0.90 [0.87-0.94]	-9.8	1.07 [0.91-1.26]	7.2	0.74 [0.62-0.90]	-25.6
Nov	0.91 [0.88-0.95]	-8.6	0.98 [0.94-1.02]	-2.0	0.96 [0.93-1.01]	-3.5	0.98 [0.94-1.02]	-1.7	1.10 [0.93-1.31]	10.3	1.16 [0.98-1.37]	15.5
Dec	1.19 [1.15-1.23]	19.1	1.20 [1.16-1.25]	20.5	1.17 [1.12-1.22]	16.7	1.10 [1.05-1.15]	9.9	1.12 [0.92-1.35]	11.9	1.22 [1.01-1.47]	21.9
**Age, years****												
18-49	1.00 [0.96-1.03]	-0.5	0.99 [0.95-1.03]	-1.1	0.97 [0.93-1.01]	-3.2	0.99 [0.95-1.03]	-1.1	0.88 [0.75-1.04]	-11.7	0.96 [0.82-1.12]	-3.8
50-69	0.94 [0.92-0.96]	-5.9	0.96 [0.95-0.98]	-3.7	0.96 [0.94-0.97]	-4.5	0.98 [0.96-1.00]	-2.4	0.97 [0.89-1.05]	-3.3	0.98 [0.90-1.07]	-2.0
70+	0.98 [0.96-1.00]	-2.0	1.02 [1.00-1.03]	1.7	0.96 [0.94-0.97]	-4.4	0.97 [0.95-0.98]	-3.3	1.06 [0.98-1.15]	6.0	1.07 [0.99-1.16]	7.2
**Sex*****												
Women	0.97 [0.95-0.98]	-3.4	0.99 [0.97-1.01]	-1.0	0.95 [0.93-0.97]	-4.9	0.97 [0.96-0.99]	-2.7	1.00 [0.92-1.07]	-0.4	1.01 [0.93-1.08]	0.5
Men	0.97 [0.95-0.98]	-3.4	1.00 [0.98-1.01]	-0.4	0.96 [0.95-0.98]	-3.8	0.97 [0.96-0.99]	-2.8	1.00 [0.93-1.08]	0.0	1.03 [0.96-1.12]	3.4
**Type of cancer**												
Breast (female)***	0.96 [0.93-0.99]	-4.4	0.98 [0.95-1.01]	-2.0	0.96 [0.93-0.99]	-3.8	1.00 [0.97-1.03]	0.0	1.00 [0.88-1.15]	0.2	1.00 [0.88-1.15]	0.3
Prostate***	0.95 [0.92-0.98]	-4.9	0.97 [0.94-1.00]	-3.1	0.92 [0.89-0.95]	-7.9	0.94 [0.91-0.97]	-6.4	1.15 [0.99-1.34]	15.3	1.25 [1.08-1.44]	24.6
Melanoma*	0.99 [0.94-1.03]	-1.4	1.05 [1.01-1.10]	5.0	0.87 [0.83-0.92]	-12.6	1.00 [0.95-1.06]	0.3	0.87 [0.62-1.21]	-13.4	0.98 [0.71-1.35]	-1.8
Colon*	0.89 [0.85-0.92]	-11.4	0.91 [0.88-0.95]	-8.8	1.00 [0.96-1.05]	0.2	1.02 [0.97-1.07]	2.1	0.74 [0.60-0.90]	-26.5	1.00 [0.84-1.20]	0.4
Rectum*	0.84 [0.79-0.89]	-16.3	0.89 [0.84-0.94]	-11.1	0.96 [0.91-1.03]	-3.6	1.02 [0.96-1.08]	2.0	0.88 [0.63-1.25]	-11.6	1.17 [0.86-1.60]	17.3
Lung*	1.00 [0.97-1.04]	0.4	1.01 [0.98-1.04]	0.7	0.96 [0.92-1.00]	-3.7	0.92 [0.89-0.96]	-7.5	1.02 [0.87-1.21]	2.2	0.89 [0.75-1.06]	-10.9
Kidney*	1.00 [0.94-1.08]	0.5	1.06 [0.99-1.14]	6.2	0.95 [0.88-1.02]	-5.4	0.97 [0.90-1.04]	-3.1	1.22 [0.93-1.60]	22.2	1.20 [0.91-1.57]	19.5

*adjusted for age, sex. ** adjusted for sex. *** adjusted for age.

Continued.

	NORWAY				SWEDEN			
	**2020**		**2021**		**2020**		**2021**	
	**IRR (95% CI)**	**PC %**	**IRR (95% CI)**	**PC %**	**IRR (95% CI)**	**PC %**	**IRR (95% CI)**	**PC %**
**Total (annual)***	0.98 [0.97-0.99]	-2.3	0.99 [0.98-1.00]	-1.0	0.92 [0.92-0.93]	-7.6	0.96 [0.96-0.97]	-3.6
**Diagnosis period (quarterly)***								
Q1: Jan-Mar	0.99 [0.97-1.02]	-0.8	0.98 [0.95-1.00]	-2.3	0.97 [0.95-0.98]	-3.4	0.91 [0.90-0.93]	-8.6
Q2: Apr-Jun	0.88 [0.86-0.90]	-12.2	0.99 [0.96-1.01]	-1.3	0.78 [0.77-0.80]	-21.7	1.00 [0.98-1.01]	-0.4
Q3: Jul-Sep	1.01 [0.99-1.04]	1.5	1.01 [0.98-1.04]	1.0	0.97 [0.95-0.99]	-3.0	0.98 [0.96-1.00]	-1.8
Q4: Oct-Dec	1.03 [1.00-1.05]	2.8	0.99 [0.96-1.01]	-1.3	0.98 [0.96-1.00]	-2.2	0.97 [0.95-0.99]	-3.1
**Diagnosis period (monthly)***								
Jan	1.02 [0.98-1.06]	2.1	0.90 [0.86-0.94]	-10.3	1.00 [0.97-1.03]	0.4	0.80 [0.77-0.83]	-20.2
Feb	1.04 [1.00-1.09]	4.0	1.01 [0.97-1.05]	0.8	0.99 [0.96-1.02]	-1.2	0.94 [0.91-0.97]	-6.2
Mar	0.92 [0.88-0.96]	-8.3	1.03 [0.99-1.08]	3.3	0.91 [0.88-0.94]	-9.1	1.01 [0.98-1.04]	0.6
Apr	0.85 [0.81-0.89]	-15.3	0.99 [0.95-1.04]	-0.8	0.77 [0.74-0.79]	-23.4	0.97 [0.94-1.00]	-2.8
May	0.81 [0.77-0.85]	-19.1	0.90 [0.87-0.94]	-9.7	0.67 [0.65-0.70]	-32.7	0.92 [0.90-0.95]	-7.6
Jun	0.97 [0.94-1.02]	-2.5	1.07 [1.03-1.11]	6.6	0.93 [0.90-0.96]	-7.4	1.10 [1.07-1.14]	10.5
Jul	1.01 [0.97-1.06]	1.4	0.97 [0.93-1.02]	-2.9	0.93 [0.90-0.97]	-6.5	0.95 [0.92-0.99]	-4.7
Aug	0.97 [0.93-1.01]	-3.0	0.97 [0.93-1.01]	-3.1	0.92 [0.89-0.95]	-7.7	0.97 [0.94-1.00]	-3.0
Sep	1.06 [1.02-1.10]	5.8	1.08 [1.04-1.13]	8.0	1.04 [1.01-1.08]	4.4	1.02 [0.99-1.05]	1.8
Oct	1.00 [0.96-1.04]	-0.0	0.89 [0.86-0.93]	-10.6	0.94 [0.91-0.96]	-6.4	0.89 [0.86-0.92]	-11.3
Nov	1.01 [0.97-1.06]	1.3	0.99 [0.95-1.03]	-0.7	0.97 [0.94-1.00]	-2.9	1.00 [0.97-1.03]	0.0
Dec	1.08 [1.03-1.13]	8.0	1.09 [1.05-1.14]	9.2	1.04 [1.00-1.07]	3.8	1.03 [1.00-1.07]	3.4
**Age, years****								
18-49	1.00 [0.97-1.04]	0.3	0.96 [0.92-0.99]	-4.3	0.96 [0.93-0.99]	-4.0	0.95 [0.93-0.98]	-4.6
50-69	0.95 [0.93-0.97]	-5.3	0.99 [0.97-1.01]	-1.4	0.91 [0.90-0.93]	-8.7	0.94 [0.92-0.95]	-6.1
70+	1.00 [0.98-1.01]	-0.4	1.00 [0.98-1.02]	-0.1	0.93 [0.91-0.94]	-7.5	0.98 [0.97-1.00]	-1.6
**Sex*****								
Women	0.97 [0.96-0.99]	-2.8	1.01 [0.99-1.03]	1.0	0.94 [0.93-0.95]	-5.9	0.99 [0.97-1.00]	-1.4
Men	0.98 [0.97-1.00]	-1.9	0.97 [0.96-0.99]	-2.7	0.91 [0.90-0.92]	-9.1	0.94 [0.93-0.96]	-5.5
**Type of cancer**								
Breast (female)***	0.93 [0.90-0.97]	-6.7	1.08 [1.04-1.12]	7.7	0.93 [0.91-0.95]	-7.2	1.03 [1.01-1.06]	3.3
Prostate***	0.96 [0.93-0.99]	-3.7	0.96 [0.93-0.99]	-3.6	0.81 [0.79-0.83]	-19.0	0.90 [0.88-0.92]	-9.9
Melanoma*	1.00 [0.96-1.05]	0.1	1.02 [0.98-1.06]	1.8	1.00 [0.97-1.04]	0.2	1.08 [1.05-1.12]	8.4
Colon*	0.97 [0.93-1.00]	-3.3	0.98 [0.95-1.02]	-1.7	0.99 [0.95-1.02]	-1.4	1.04 [1.01-1.07]	3.7
Rectum*	1.03 [0.96-1.09]	2.6	0.94 [0.88-1.00]	-6.0	0.93 [0.89-0.98]	-7.0	0.99 [0.95-1.04]	-0.6
Lung*	0.96 [0.92-1.00]	-4.2	0.98 [0.94-1.02]	-2.2	N/A	N/A	N/A	N/A
Kidney*	0.94 [0.87-1.01]	-6.0	0.95 [0.89-1.03]	-4.7	N/A	N/A	N/A	N/A

*adjusted for age, sex. ** adjusted for sex. *** adjusted for age.

Supplemental Table S5. Age and sex-adjusted incidence rate ratios (IRR) comparing 2020 and 2021 versus 2017-2019, by diagnostic period (quarterly) and cancer site per country. Corresponding to Figure 2.

	DENMARK				FINLAND				ICELAND			
	2020		2021		2020		2021		2020		2021	
	IRR (95% CI)	PC %	IRR (95% CI)	PC %	IRR (95% CI)	PC %	IRR (95% CI)	PC %	IRR (95% CI)	PC %	IRR (95% CI)	PC %
**Breast**												
Q1	0.93 [0.87-1.00]	-6.8	0.94 [0.88-1.01]	-5.6	0.98 [0.92-1.05]	-1.5	1.00 [0.94-1.07]	0.5	0.94 [0.72-1.24]	-5.9	0.96 [0.74-1.26]	-3.7
Q2	0.82 [0.77-0.88]	-17.8	0.97 [0.91-1.04]	-2.9	0.85 [0.80-0.91]	-14.8	1.03 [0.97-1.09]	2.8	0.75 [0.56-0.99]	-25.5	0.96 [0.74-1.24]	-4.3
Q3	1.02 [0.95-1.09]	1.8	0.97 [0.91-1.04]	-2.7	1.00 [0.93-1.06]	-0.4	0.98 [0.92-1.05]	-1.6	1.16 [0.88-1.54]	16.1	1.09 [0.82-1.45]	8.9
Q4	1.05 [0.98-1.11]	4.5	1.03 [0.97-1.09]	2.7	1.01 [0.96-1.08]	1.5	0.98 [0.93-1.05]	-1.6	1.21 [0.94-1.56]	21.3	1.02 [0.78-1.34]	2.3
**Prostate**												
Q1	0.98 [0.92-1.05]	-1.6	0.98 [0.92-1.05]	-1.8	0.92 [0.87-0.98]	-7.6	0.86 [0.81-0.91]	-14.3	0.95 [0.71-1.29]	-4.5	1.11 [0.84-1.47]	11.1
Q2	0.73 [0.68-0.79]	-26.6	1.01 [0.95-1.08]	1.4	0.80 [0.75-0.85]	-20.1	0.96 [0.90-1.02]	-3.8	1.39 [1.04-1.86]	39.4	1.36 [1.01-1.81]	35.6
Q3	1.08 [1.01-1.16]	8.2	0.87 [0.81-0.94]	-12.8	0.95 [0.89-1.01]	-5.1	0.92 [0.86-0.98]	-7.9	1.09 [0.79-1.51]	9.3	1.19 [0.87-1.62]	18.8
Q4	1.00 [0.94-1.06]	-0.0	1.00 [0.94-1.06]	-0.4	1.00 [0.94-1.06]	0.0	0.98 [0.92-1.04]	-2.1	1.20 [0.90-1.60]	20.0	1.34 [1.02-1.76]	34.1
**Melanoma**												
Q1	1.03 [0.93-1.13]	2.7	0.91 [0.83-1.01]	-8.7	0.95 [0.85-1.07]	-4.9	1.01 [0.90-1.13]	1.0	0.73 [0.39-1.37]	-27.1	0.65 [0.34-1.26]	-34.7
Q2	0.90 [0.83-0.98]	-9.9	1.01 [0.93-1.10]	1.3	0.65 [0.58-0.73]	-35.1	0.94 [0.85-1.05]	-5.6	0.88 [0.40-1.94]	-12.2	0.86 [0.39-1.90]	-14.1
Q3	1.06 [0.98-1.16]	6.4	1.17 [1.07-1.26]	16.6	0.96 [0.86-1.07]	-3.6	1.05 [0.95-1.17]	5.3	1.01 [0.52-1.95]	0.8	1.23 [0.67-2.26]	23.2
Q4	0.97 [0.89-1.05]	-3.3	1.08 [1.00-1.17]	8.1	0.97 [0.87-1.08]	-3.0	1.01 [0.91-1.13]	1.2	0.90 [0.47-1.73]	-9.8	1.25 [0.70-2.21]	24.9
**Colon**												
Q1	0.85 [0.78-0.92]	-15.0	0.82 [0.76-0.89]	-17.9	0.96 [0.87-1.06]	-3.9	0.96 [0.87-1.05]	-4.3	0.78 [0.52-1.16]	-22.2	0.85 [0.58-1.25]	-14.6
Q2	0.78 [0.72-0.85]	-22.0	0.89 [0.83-0.97]	-10.7	0.92 [0.84-1.02]	-7.5	1.04 [0.95-1.14]	3.6	0.58 [0.38-0.89]	-41.9	0.79 [0.54-1.15]	-20.9
Q3	0.97 [0.89-1.05]	-3.2	0.92 [0.85-1.00]	-7.6	1.06 [0.96-1.16]	5.6	1.01 [0.92-1.11]	1.4	0.91 [0.60-1.36]	-9.4	1.31 [0.92-1.86]	30.8
Q4	0.95 [0.88-1.02]	-5.2	1.00 [0.93-1.08]	0.5	1.07 [0.97-1.17]	6.8	1.08 [0.98-1.18]	7.6	0.71 [0.48-1.05]	-28.6	1.11 [0.80-1.53]	10.8
**Lung**												
Q1	1.11 [1.04-1.19]	11.2	0.99 [0.92-1.05]	-1.4	0.95 [0.88-1.04]	-4.6	0.93 [0.86-1.01]	-6.7	0.97 [0.69-1.36]	-3.0	0.71 [0.49-1.04]	-28.6
Q2	0.89 [0.84-0.95]	-10.7	0.97 [0.91-1.03]	-3.1	0.90 [0.83-0.98]	-9.6	0.90 [0.83-0.98]	-10.3	1.14 [0.83-1.57]	13.8	0.90 [0.63-1.27]	-10.4
Q3	1.04 [0.98-1.11]	3.9	1.05 [0.98-1.12]	4.8	1.01 [0.93-1.10]	0.8	0.94 [0.86-1.02]	-6.0	0.93 [0.67-1.30]	-7.2	0.90 [0.65-1.26]	-9.6
Q4	0.98 [0.92-1.05]	-1.7	1.02 [0.96-1.09]	2.2	0.97 [0.90-1.06]	-2.6	0.91 [0.83-0.99]	-9.5	1.06 [0.76-1.48]	6.2	1.06 [0.76-1.47]	5.6

	NORWAY				SWEDEN			
	2020		2021		2020		2021	
	IRR (95% CI)	PC %	IRR (95% CI)	PC %	IRR (95% CI)	PC %	IRR (95% CI)	PC %
**Breast**								
Q1	0.99 [0.92-1.06]	-1.1	0.99 [0.93-1.07]	-0.5	0.98 [0.94-1.03]	-1.8	0.98 [0.93-1.02]	-2.4
Q2	0.74 [0.68-0.79]	-26.5	1.11 [1.04-1.18]	10.9	0.75 [0.71-0.79]	-25.3	1.08 [1.03-1.13]	7.5
Q3	1.02 [0.94-1.10]	1.6	1.14 [1.05-1.22]	13.6	0.99 [0.94-1.04]	-1.2	1.04 [0.99-1.09]	3.8
Q4	1.01 [0.94-1.08]	1.1	1.08 [1.01-1.16]	8.3	1.00 [0.95-1.05]	0.1	1.05 [1.00-1.10]	4.5
**Prostate**								
Q1	0.96 [0.90-1.02]	-4.1	0.94 [0.88-0.99]	-6.3	0.93 [0.89-0.97]	-7.0	0.78 [0.75-0.82]	-21.9
Q2	0.86 [0.81-0.92]	-13.7	0.94 [0.88-1.00]	-5.7	0.61 [0.58-0.64]	-39.1	0.93 [0.89-0.97]	-7.3
Q3	0.97 [0.90-1.04]	-3.3	1.04 [0.97-1.11]	3.5	0.85 [0.81-0.90]	-14.5	0.95 [0.90-1.00]	-5.2
Q4	1.06 [1.00-1.13]	6.1	0.96 [0.90-1.02]	-4.4	0.84 [0.80-0.88]	-15.8	0.97 [0.93-1.01]	-3.1
**Melanoma**								
Q1	0.99 [0.91-1.09]	-0.6	0.95 [0.86-1.04]	-5.4	1.11 [1.04-1.19]	11.3	1.02 [0.96-1.10]	2.3
Q2	0.90 [0.83-0.98]	-10.0	1.00 [0.92-1.09]	0.1	0.84 [0.79-0.90]	-15.6	1.08 [1.02-1.15]	8.2
Q3	1.14 [1.04-1.24]	13.8	1.09 [1.00-1.19]	8.9	1.04 [0.97-1.11]	4.2	1.13 [1.06-1.21]	13.0
Q4	0.99 [0.91-1.09]	-0.8	1.04 [0.95-1.13]	3.9	1.03 [0.97-1.10]	3.4	1.10 [1.03-1.17]	10.0
**Colon**								
Q1	0.96 [0.89-1.03]	-4.4	1.02 [0.95-1.10]	2.3	1.00 [0.94-1.06]	-0.2	0.97 [0.91-1.04]	-2.8
Q2	0.93 [0.86-1.00]	-7.4	0.97 [0.90-1.04]	-3.2	0.84 [0.78-0.90]	-16.2	1.06 [1.00-1.13]	6.3
Q3	0.97 [0.90-1.05]	-3.0	0.95 [0.87-1.02]	-5.5	1.00 [0.93-1.06]	-0.4	1.04 [0.98-1.11]	3.9
Q4	1.02 [0.94-1.10]	1.6	1.00 [0.92-1.07]	-0.5	1.11 [1.04-1.18]	11.1	1.08 [1.01-1.14]	7.6
**Lung**								
Q1	1.02 [0.95-1.10]	2.3	0.98 [0.91-1.06]	-2.1	N/A	N/A	N/A	N/A
Q2	0.81 [0.75-0.88]	-18.6	0.98 [0.91-1.06]	-2.0	N/A	N/A	N/A	N/A
Q3	0.98 [0.90-1.06]	-2.2	0.96 [0.88-1.03]	-4.4	N/A	N/A	N/A	N/A
Q4	1.02 [0.95-1.10]	2.1	1.00 [0.92-1.08]	-0.3	N/A	N/A	N/A	N/A

All estimates adjusted for age and sex (except breast and prostate cancer, which were only adjusted for age). For Finland: Prostate, rectum, lung, kidney were only adjusted by age in 2 categories (18-69 and 70+).

Supplemental Table S6. Age and sex-adjusted stage-specific incidence rate ratios (IRR) of annual rates 2020 and 2021 vs pre-pandemic 2017-2019 per country. Corresponding to Figure 4.

	DENMARK				ICELAND				NORWAY				SWEDEN			
	2020 IRR (95% CI)	2020 PC %	2021 IRR (95% CI)	2021 PC %	2020 IRR (95% CI)	2020 PC %	2021 IRR (95% CI)	2021 PC %	2020 IRR (95% CI)	2020 PC %	2021 IRR (95% CI)	2021 PC %	2020 IRR (95% CI)	2020 PC %	2021 IRR (95% CI)	2021 PC %
**Breast**																
Stage I	0.93 [0.88-0.97]	-7.3	0.96 [0.92-1.01]	-3.6	1.02 [0.84-1.25]	2.5	1.02 [0.84-1.25]	2.1	0.87 [0.82-0.92]	-13.2	1.06 [1.01-1.12]	6.5	0.87 [0.84-0.91]	-12.5	0.96 [0.93-0.99]	-4.0
Stage II	1.01 [0.95-1.06]	0.6	1.05 [0.99-1.11]	4.9	0.94 [0.74-1.20]	-5.7	1.06 [0.84-1.34]	6.2	1.01 [0.95-1.07]	0.7	1.11 [1.05-1.17]	10.7	0.95 [0.91-0.99]	-5.4	1.08 [1.04-1.12]	8.2
Stage III	1.00 [0.89-1.12]	-0.3	1.09 [0.97-1.21]	8.6	0.97 [0.62-1.53]	-3.0	1.03 [0.66-1.59]	2.5	0.85 [0.76-0.96]	-14.8	0.95 [0.85-1.06]	-5.1	0.92 [0.81-1.04]	-8.4	1.07 [0.95-1.20]	6.6
Stage IV	1.02 [0.85-1.21]	1.7	1.00 [0.84-1.19]	0.4	0.87 [0.47-1.61]	-13.3	0.78 [0.41-1.48]	-21.7	1.02 [0.82-1.27]	2.0	1.01 [0.81-1.25]	0.5	0.93 [0.81-1.06]	-7.0	1.05 [0.93-1.20]	5.4
Stage miss. %	9.5		7.9		9.6		5.9		4.7		4.6		7.3		6.7	
**Prostate**																
Stage I	0.94 [0.88-1.01]	-5.8	1.00 [0.94-1.07]	-0.1	1.72 [1.37-2.17]	72.1	1.66 [1.32-2.09]	66.0	1.05 [0.98-1.12]	5.0	1.17 [1.10-1.24]	16.8	0.77 [0.74-0.80]	-23.1	0.86 [0.83-0.89]	-13.8
Stage II	0.96 [0.89-1.02]	-4.3	0.96 [0.90-1.03]	-4.2	0.70 [0.48-1.01]	-30.2	0.89 [0.64-1.24]	-10.8	0.93 [0.88-0.99]	-7.0	0.92 [0.87-0.98]	-7.7	0.81 [0.78-0.85]	-18.7	0.87 [0.84-0.91]	-12.9
Stage III	0.99 [0.91-1.08]	-1.0	1.03 [0.95-1.12]	3.3	1.05 [0.73-1.49]	4.5	1.14 [0.81-1.60]	13.8	1.03 [0.96-1.09]	2.7	0.96 [0.90-1.02]	-4.4	0.81 [0.76-0.87]	-18.7	0.94 [0.88-1.00]	-6.2
Stage IV	1.20 [1.12-1.29]	19.8	1.28 [1.19-1.37]	27.7	0.34 [0.19-0.61]	-65.9	0.84 [0.57-1.24]	-16.2	1.11 [1.02-1.21]	10.8	1.10 [1.02-1.20]	10.4	0.82 [0.75-0.90]	-17.6	1.09 [1.01-1.19]	9.5
Stage miss %	9.9		7.8		14.9		10.4		7.3		6.8		6.0		5.1	
**Melanoma**																
Stage I	0.93 [0.88-0.98]	-7.0	1.03 [0.98-1.08]	2.6	0.87 [0.57-1.34]	-13.0	0.89 [0.59-1.33]	-11.3	1.01 [0.95-1.06]	0.7	1.04 [0.99-1.10]	4.0	1.08 [1.04-1.12]	8.0	1.17 [1.13-1.22]	17.1
Stage II	1.08 [0.97-1.21]	8.2	1.02 [0.91-1.14]	2.1	0.97 [0.47-1.97]	-3.4	0.93 [0.47-1.86]	-6.6	0.96 [0.87-1.06]	-4.3	0.98 [0.89-1.08]	-2.1	1.20 [1.11-1.29]	19.6	1.19 [1.11-1.28]	19.0
Stage III	1.45 [1.25-1.68]	45.1	1.47 [1.27-1.70]	46.8	1.18 [0.46-3.05]	18.3	1.05 [0.41-2.70]	4.8	1.16 [0.98-1.37]	15.8	1.04 [0.87-1.23]	3.5	0.82 [0.62-1.09]	-17.8	1.05 [0.81-1.36]	5.3
Stage IV	1.20 [0.94-1.52]	19.5	1.18 [0.93-1.50]	18.0	NA	NA	NA	NA	1.58 [0.84-2.97]	57.8	0.92 [0.43-1.96]	-7.6	0.73 [0.45-1.17]	-27.2	1.34 [0.91-1.95]	33.5
Stage miss %	NA		NA		2.3		2.0		4.4		4.4		5.9		6.8	
**Colon**																
Stage I	0.95 [0.87-1.03]	-5.4	0.90 [0.82-0.98]	-10.4	NA	NA	NA	NA	0.87 [0.79-0.96]	-13.0	0.91 [0.83-1.00]	-9.2	0.93 [0.86-1.00]	-7.3	0.99 [0.92-1.07]	-1.1
Stage II	0.88 [0.81-0.96]	-12.2	0.95 [0.88-1.03]	-4.5	NA	NA	NA	NA	0.97 [0.90-1.04]	-3.0	1.00 [0.94-1.07]	0.3	0.91 [0.84-0.98]	-9.5	1.00 [0.93-1.07]	0.0
Stage III	0.94 [0.87-1.02]	-5.8	0.98 [0.91-1.05]	-2.1	NA	NA	NA	NA	0.98 [0.91-1.06]	-1.8	0.99 [0.92-1.07]	-0.5	1.03 [0.97-1.10]	3.4	1.12 [1.05-1.18]	11.7
Stage IV	0.93 [0.85-1.01]	-7.4	1.01 [0.93-1.10]	1.3	NA	NA	NA	NA	0.99 [0.90-1.09]	-0.8	0.95 [0.87-1.05]	-4.7	1.05 [0.98-1.12]	4.9	0.98 [0.91-1.05]	-2.1
Stage miss %	7.2		6.0		NA		NA		12.1		12.2		14.4		14.9	
**Lung**																
Stage I	1.10 [1.02-1.18]	9.9	1.37 [1.29-1.46]	37.3	NA	NA	NA	NA	1.05 [0.96-1.13]	4.5	1.07 [0.99-1.16]	7.1	N/A	N/A	N/A	N/A
Stage II	1.02 [0.91-1.13]	1.5	0.92 [0.82-1.02]	-8.4	NA	NA	NA	NA	0.81 [0.71-0.94]	-18.5	0.91 [0.80-1.04]	-8.9	N/A	N/A	N/A	N/A
Stage III	0.91 [0.85-0.98]	-8.6	0.94 [0.87-1.01]	-6.5	NA	NA	NA	NA	0.96 [0.87-1.05]	-4.3	0.96 [0.88-1.05]	-4.0	N/A	N/A	N/A	N/A
Stage IV	1.01 [0.96-1.05]	0.7	0.99 [0.94-1.03]	-1.3	NA	NA	NA	NA	1.12 [1.06-1.20]	12.4	1.14 [1.07-1.21]	13.7	N/A	N/A	N/A	N/A
Stage miss %	5.1		1.4		NA		NA		11.7		11.7		N/A		N/A	

For Denmark: Only adjusted by age in 2 categories (18-69, 70+) for sites: Prostate stage II, III, IV; Melanoma stage III, IV; Lung stage I, II, III.

Supplemental Figure S1. Weekly incidence (N) of COVID-19 per country 2020-2021. Source: www.ourworldindata.com .

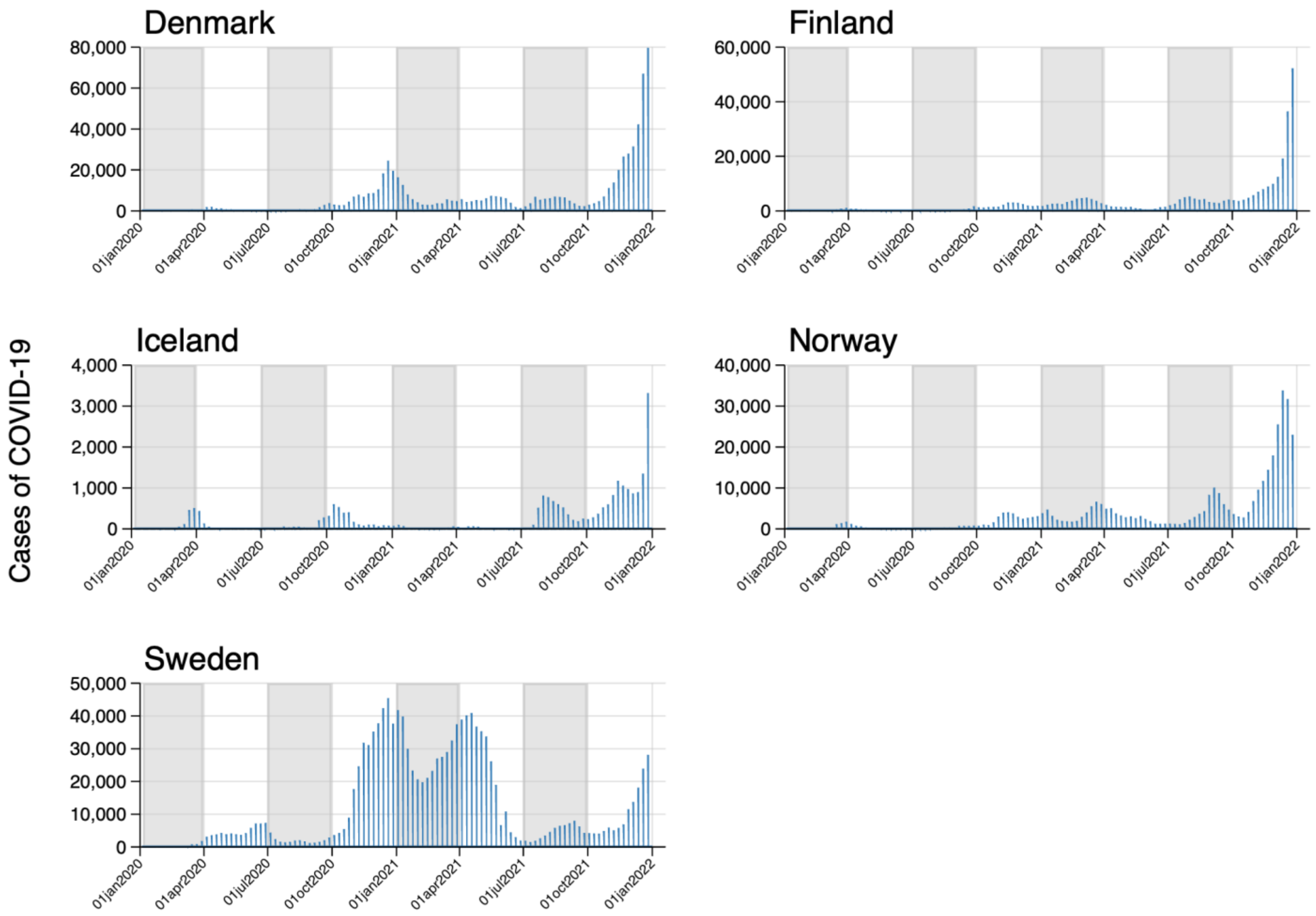

Supplement: Changes in cancer incidence and stage during the COVID-19 pandemic in 2020–2021 in the Nordic countries [file AO-64-42079-s1.pdf]
